# A clinical study on ozone autohemotherapy for the treatment of acute ischemic stroke

**DOI:** 10.3389/fmed.2025.1595568

**Published:** 2025-07-25

**Authors:** Heyun Cheng, Rui Lu, Juan Du, Xingjuan Zhao, Zhijiang Zhuang

**Affiliations:** Department of Neurology, The Fifth Affiliated Hospital of Zhengzhou University, Zhengzhou, China

**Keywords:** ozonated autohemotherapy, acute cerebral infarction, oxidative stress damage, ozone oxidative preconditioning, hypoxia-inducible factor

## Abstract

**Background:**

Ozonated autohemotherapy is a therapeutic method that utilizes the contact of medical ozone with blood to achieve the effect of ozone oxidative preconditioning, and then re-infuses it back into the human body. It has the functions of alleviating oxidative stress damage, reducing neuroexcitation toxicity, and mitigating cellular edema and inflammatory responses. Cerebral infarction has a significant social hazard, and there are multiple treatment methods available. There have been reports of treating cerebral infarction with ozonated autohemotherapy, but the efficacy and mechanism of action are not yet clear. The background now includes information about the proteins measured, specifically neuron-specific enolase (NSE) and S100β protein, which are markers of neuronal cell damage.

**Methods:**

A randomized controlled study was conducted, enrolling 62 patients with acute cerebral infarction to investigate the therapeutic effect of ozonated autohemotherapy on acute cerebral infarction. The study included three groups: a control group, an oxygen placebo group, and an ozone therapy group. The intervention was administered over a period of 5 days, with patients undergoing treatment within 24 h of symptom onset. Inclusion criteria comprised patients diagnosed with acute cerebral infarction, aged >18 years, with an NIHSS score between 4 and 15. Exclusion criteria included other neurological disorders, severe infectious diseases, and contraindications for ozone therapy. The efficacy was primarily evaluated through neurological function scoring, motor function scoring, cognitive scoring, and the detection of neuron-specific enolase (NSE) and S100β protein (A neurotrophic factor), which are markers of neuronal cell damage. The two genes involved in the study are HIF-1 and Nrf-2. The mechanism of ozonated autohemotherapy in treating acute cerebral infarction was also explored by detecting these two genes and three indicators related to oxidative stress in the body.

**Results:**

Ozonated autohemotherapy significantly improved the prognosis of acute cerebral infarction, with NIHSS scores decreasing by 30%, Barthel Index scores increasing by 25%, and MoCA scores improving by 20% compared to the control group. Levels of NSE and S100-*β* protein were reduced by 25 and 30%, respectively, compared to baseline. In the ozone treatment group, superoxide dismutase (SOD) and malondial Dehyde (MDA) levels decreased, while glutathione peroxidase (GSH-Px) levels increased, and both HIF-1 and Nrf-2 levels were elevated compared to before. Moreover, it did not increase the risk of heart, liver, and kidney damage.

**Conclusion:**

It suggests that ozone may improve oxidative stress by regulating HIF-1 and Nrf-2, reduce the oxidative stress response and inflammatory damage caused by ischemia of neuronal cells in acute cerebral infarction, thereby improving neurological function and prognosis. The treatment method is safe in the short term. Long-term safety requires further research.

## 1 Introduction

Cerebral infarction, resulting from sudden impairment of cerebral blood circulation due to various factors, is characterized by high incidence, disability, and mortality rates. It is prone to recurrence and adversely impacts the quality of life for patients and their families, imposing substantial economic and emotional burdens on society (1–4). According to the Global Burden of Disease Study 2019 (GBD 2019) (5), stroke ranks as the second leading cause of death (COD) and the third leading cause of disability-adjusted life years (DALYs) worldwide in 2019 (6). In China, stroke accounted for 33.4% of global stroke deaths, with a significantly higher age-standardized mortality rate (5.2019 times higher) compared to the global average (127.2 vs. 84.2/100,000). Given aging societies globally, timely and effective treatment for patients with acute stroke has become a pressing. Thrombolysis is the primary treatment for acute cerebral infarction within the ultra-early stage (0–4.5 h) (7–9). Mechanical thrombectomy has also emerged as a highly effective treatment option for acute ischemic stroke, especially for patients with large vessel occlusions. However, if the thrombolysis window is missed or the stroke progresses post-thrombolysis without feasible intra-arterial treatments like bridging therapy, or if specialized equipment and expertise for mechanical thrombectomy are unavailable, options for effective acute ischemic stroke treatment are limited (10, 11). This limitation can lead to permanent functional impairments affecting speech, sensation, and limb movement, thereby severely impacting patient behavioral and social capabilities (12, 13).

Ischemic damage to brain cells results in the significant release of excitotoxic amino acids triggering immediate oxidative stress response and neuroinflammation, leading to secondary brain damage around the core infarction zone (14–16). Reactive oxygen species (ROS) generated by oxidative stress can directly compromise the blood–brain barrier (BBB) integrity and recruit peripheral immune cells, initiating inflammatory cascades that exacerbate ischemic and surrounding brain tissue (17–19). Thrombolysis and mechanical thrombectomy in the ultra-early stroke stage can restore reperfusion to the ischemic penumbra (ischemia–reperfusion, I/R), representing crucial stroke treatments (20). However, I/R induces injury responses including oxidative stress, excitotoxicity, inflammation, autophagy, and edema, further complicating disease progression (21–25).

Ozone, a highly oxidative gas harmful upon direct contact with the human body (26), has been Ozone has a historical application in surgery for sterilization and disinfection (27–29). However, due to its strong oxidizing properties, prolonged exposure to high doses of ozone can cause burns and even carcinogenic effects when in contact with skin and mucous membranes (30). To mitigate these risks, ozone has been refined into medical ozone—a mixture with oxygen that induces moderate oxidative stress and activates antioxidant pathways, thereby proving safe and effective for medical use (31–35).

Medical ozone, upon contact with blood, initiates ozone oxidation pre-treatment, generating ROS like superoxide anions and hydrogen peroxide. These substances partially inhibit oxidative stress reactions, enhance endogenous antioxidant production, counteract hypoxia and stress from ischemic damage, and restore balance between endogenous antioxidants and prooxidants (36, 37). Moreover, ozone boosts endogenous free radical scavenging enzyme activity, bolstering free radical removal and alleviating oxidative stress (38).

Since the 1980s, ozone autohemotherapy has been progressively used in stroke treatment, adhering to safe dosages within the range of plasma total antioxidant system (TAS) buffering capacity (20–80 μg/mL O_3_, not exceeding 160 μg/mL) (26, 35, 39). In other studies, medical ozone has typically been administered at a dose of 5–20 mL, ranging from 3 to 5 times per day, demonstrating both safety and efficacy in various clinical settings. In China, ozone autohemotherapy has been used in the clinical treatment of cerebrovascular diseases since the 1990s, establishing a reputation for safety and efficacy (40, 41). This study aims to further validate the application of ozone autohemotherapy in acute cerebrovascular diseases through clinical trials, exploring its efficacy and potential mechanisms in the acute phase of ischemic stroke from the aspects of therapeutic efficacy, antioxidant capacity, and pathways.

## 2 Materials and methods

### 2.1 Study population

This study utilized a randomized controlled design involving 62 patients with acute ischemic stroke admitted to the Department of Neurology between August 2020 to October 2022, meeting specific inclusion and exclusion criteria. The cohort comprised 40 males and 22 females, aged between 37 and 85 years, with an average age of (64.13 ± 4.32) years. The diagnosis of acute ischemic stroke followed the “Chinese Guidelines for the Diagnosis and Treatment of Acute Ischemic Stroke 2018”, Confirmed by cranial CT or MRI examination, first-ever stroke, hospital admission within <24 h of symptom onset, age >18 years, NIHSS score between 4 and 15, TOAST classification as large artery atherosclerosis, complete clinical records, minimum follow-up period of ≥3 months post-discharge, willingness to undergo brachial blood puncture treatment, and provision of informed consent. Exclusion criteria encompassed other neurological disorders, cerebral infarction caused by vertebral-basilar artery disease, cardioembolic stroke, or large-vessel vasculitis, acute or chronic infectious diseases, autoimmune or hematologic disorders, malignant tumors, mental abnormalities, severe heart, liver, or kidney dysfunction, intracerebral hemorrhage, and craniocerebral surgery history. Contraindications for ozone therapy included hyperthyroidism, favism, severe coagulation disorders, and abnormal puncture site barriers.

### 2.2 Methods

The study included 62 patients (approximately 20 per group), with a sample size calculation based on effect sizes from previous studies and statistical power analysis. The sample size was determined to ensure sufficient statistical power to detect significant differences between groups.

Patients were randomly divided into three groups using a stratified randomization method, and the study was conducted as a double-blind trial (the placebo and ozone therapy groups were indistinguishable to patients during the treatment process). Group A served as the control group, Group B received an oxygen placebo, and Group C underwent ozone therapy. All patients received standard treatments, including antiplatelet aggregation therapy (aspirin 200 mg, Bayer Health Care Co., Ltd.), plaque stabilizing lipid-lowering therapy (rosuvastatin calcium tablets 20 mg, Zhejiang Haizheng Pharmaceutical Co., Ltd.), and conventional therapies to enhance cerebral metabolism and neuro-nutrition. In addition to these treatments, the placebo group received a 5-day intravenous infusion of 100 mL of 45% oxygen mixed with 100 mL of autologous blood. The ozone therapy group received a 5-day intravenous infusion of 100 mL of 45% medical ozone mixed with 100 mL autologous blood. Ozone was prepared and blood was reinfused using a HYPER-MEDOZON medical ozone therapy device produced by Germany’s Herrmann Company. The preparation of the ozone autohemotherapy mixture involved the following steps: First, 100 mL of venous blood was drawn from each patient. This blood was then mixed with 100 mL of medical ozone gas at a concentration of 45%. The mixture was prepared immediately before infusion to ensure optimal therapeutic efficacy. The infusion was administered intravenously through a peripheral vein (usually the median cubital vein), with a total volume of 200 mL delivered over a period of 30 min. This procedure was repeated once daily for 5 consecutive days. The concentration of ozone used in the medical gas mixture was 45%, which was specifically selected to ensure both safety and efficacy based on previous studies and clinical guidelines. This concentration is crucial for reproducibility and comparison with other studies (27, 42). To ensure the quality and consistency of the ozone preparation, the following quality control measures were implemented: daily calibration of the ozone generator using a standardized calibration tool to ensure accurate ozone concentration delivery; strict adherence to aseptic techniques during blood draws and reinfusion to prevent contamination; verification of the ozone concentration in each batch of the mixture using a UV spectrophotometer to ensure accuracy; detailed documentation and daily monitoring of the treatment process for each patient; and establishment of a system for the immediate reporting and investigation of any adverse events.

Ozone therapy was initiated within 24 h of symptom onset for all patients in the ozone treatment group. The average time from symptom onset to the initiation of ozone therapy was 12 h. This timing was chosen to ensure that the therapy was administered within a clinically relevant window, maximizing its potential benefits while minimizing the risk of complications (41).

This study was approved by the Ethics Committee of ClinicalTrials, Ethics Committee approval number: NCT06525792.

### 2.3 Outcome measures

Neurological functional outcomes were assessed using the National Institutes of Health Stroke Scale (NIHSS) (43). Reductions of 20–49%, 50–89%, and ≥ 90% in NIHSS scores correspond to partial self-care with symptom disappearance, basic restoration with complete self-care and symptom disappearance, and effective restoration with complete self-cate and symptom disappearance, respectively. Neurological function was further evaluated through NIHSS scores, cerebral infarction volume, neuron-specific enolase (NSE), and S100*β* levels, indicating nerve damage. While S100β is a marker of neuronal injury, it is important to note that it is primarily associated with damage to the central nervous system rather than the peripheral nervous system. The NIHSS scale ranges up to 42 points, with ≤ 4 points indicating mild stroke and ≥21 points indicating severe stroke, assessing consciousness, gaze, limb movement, sensation, and speech (10). Head CT scans were conducted before and after the treatment period to observe cerebral infarction locations in both groups. Fasting venous blood samples of 5 mL were collected from both the control and observation groups before and after treatment. Serum was obtained after centrifugation at 3000 r/min. Furthermore, the levels of serum NSE and S100*β* protein were determined using enzyme-linked immunosorbent assay (ELISA). Cognitive function was evaluated using the Montreal Cognitive Assessment (MoCA) scale, with higher scores indicating better cognitive function. Motor function was assessed using the Barthel Index, with higher scores indicating better daily motor function. Three months post-treatment, the aforementioned scores were assessed through follow-up visits.

Laboratory tests: Before treatment (within 48 h of onset) and 1-day post-treatment completion (7 days after onset), 10 mL peripheral venous blood samples were collected from each group. The control group had peripheral venous blood collected during physical examination. Samples were centrifuged at 3,500 r/min for 10 min. Superoxide dismutase (SOD) levels were measured using chemiluminescence assay, malondialdehyde (MDA) levels via radioimmunoassay, and glutathione peroxidase (GSH-Px) levels via colorimetric assay. Serum levels of HIF-1 and Nrf-2 were determined using fluorescence polymerase chain reaction (PCR) assay, with Taq Pro Universal SYBR qPCR Master Mix (Vazyme, China) as the detection reagent. GAPDH served as the reference gene, and the relative expression levels of target genes normalized to GAPDH were calculated using the 2^(-ΔΔCt)^ method. The primer sequences used were as Table 1 follows:

**Table 1:** List of primers used for RT-PCR.

Antisense	Sense	Antisense
GAPDH	5-GGCAAGTTCAACGGCACAGT-3	5-TGGTGAAGACGCCAGTAGACTC-3
HIF-1	5-GAACGTCGAAAAGAAAAGTCTCG-3	5-CCTTATCAAGATGCGAACTCACA-3
Nrf-2	5-TCAGCGACGGAAAGAGTATGA-3	5-CCACTGGTTTCTGACTGGATGT-3

### 2.4 Follow-up

Following discharge, patients were monitored through outpatient visits and phone calls. Follow-up assessments included a re-evaluation of the Houston Intracerebral Hemorrhage Score (HIHSS) and Modified Rankin Scale (MRS) evaluation was after 3 months.

### 2.5 Statistical analysis

Data are presented as mean ± standard deviation (SD). Statistical comparisons between multiple treatment groups were conducted using the Wilcoxon test or Student’s *t*-test (R software). Spearman correlation analysis determined the correlation between gut microbiota and metabolomics. Graphs were generated using R software (version 3.5.0) and GraphPad Prism 8. Results with *p* < 0.05 were considered statistically significant.

## 3 Results

In this study, three patients underwent thrombolysis, with an average time from symptom onset to treatment of 3 h. No patients received mechanical thrombectomy. Delayed hospital arrival was a common issue, with many patients presenting beyond the optimal time window for thrombolysis (0–4.5 h) and mechanical thrombectomy (up to 24 h).

### 3.1 General information comparison

General data such as gender, age, BMI, education level, site of infarction, and comorbidities were categorized and compared between the control, placebo, and treatment groups. Statistical analysis revealed no significant differences (*p* > 0.05) between these groups, as shown in Table 2.

**Table 2:** Comparison of general information between the two patient groups.

Group	Control	O_2_-treated	O_3_-treated	*F*	*p*
Gender					
Male	13	12	13	1.000	0.3742
Female	7	8	7		
Age	62.78	64.52	67.34	2.782	0.0704
BMI (kg/m^2^)	26.45	27.71	25.82	0.2609	0.7713
Diabetes	9	10	8	0.06376	0.9383
Hypertension	7	5	8	1.000	0.3742
Hyperlipemia	5	6	7	0.07819	0.9249
Cerebral hemisphere infarction	12	13	12	0.06714	0.9351
Brain stem infarction	6	4	5	0.2556	0.7753
cerebellar infarction	2	3	3	0.1377	0.8717
Thrombolysis	1/19	1/19	1/19		

### 3.2 Comparison of three groups’ neurological function

**Figure 1:**
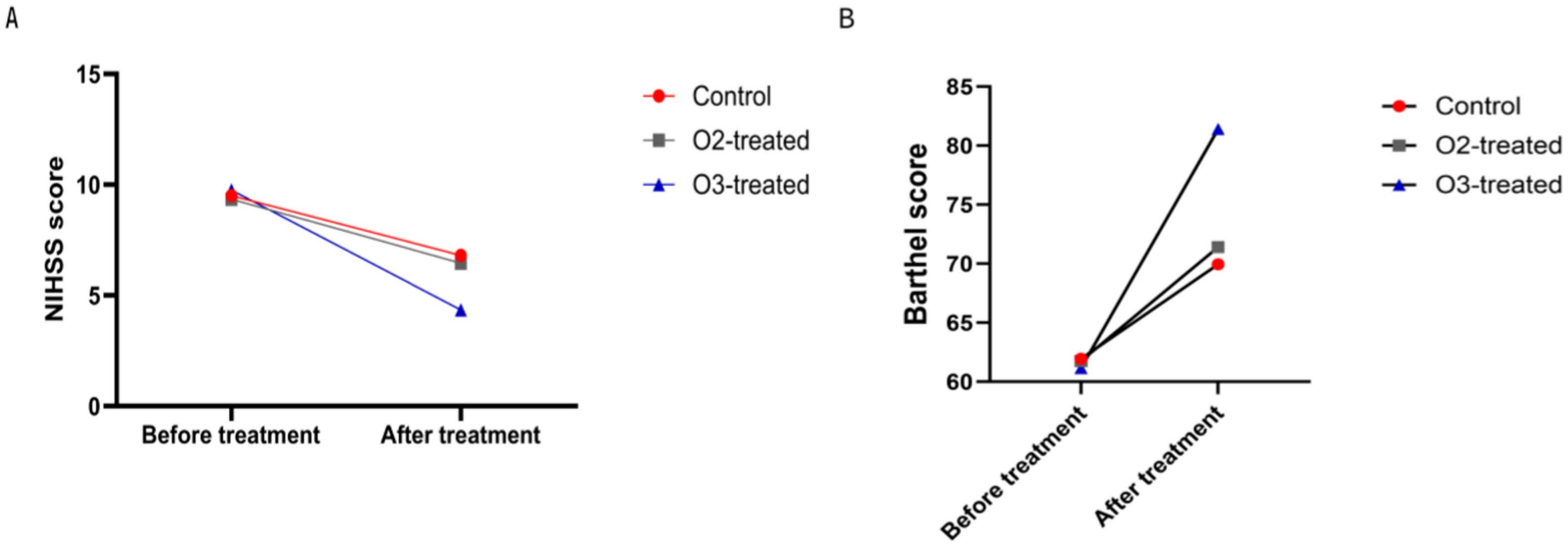
Comparison of neurological function before and after treatment. There were no significant differences in the pre-treatment NIHSS score **(A)** and motor function score (Barthel) **(B)** among the three treatment groups (*P* > 0.05). After treatment, the ozone therapy group showed a significant decrease in NIHSS score compared to before treatment, along with a significant improvement in motor function score. The control group and oxygen therapy group also exhibited a decrease in NIHSS score and an improvement in motor function score, but the degree of change in both indicators was not as significant as in the ozone therapy group (*P* < 0.05).

Pre-treatment, no significant differences in NIHSS and Barthel scores existed among the three groups (*p* > 0.05). Post-treatment, the ozone group showed a significant NIHSS reduction and Barthel improvement compared to baseline (Cohen’s d = 0.8, *p* < 0.05), indicating more effective neurological recovery than conventional therapies. The control and oxygen groups also showed score changes, but less pronounced than the ozone group (Cohen’s d = 0.3, *p* > 0.05). A one-way ANOVA was conducted to compare the NIHSS and Barthel scores across the three groups, revealing significant differences (*F* = 4.56, *p* < 0.05). *Post-hoc* analysis using Tukey’s HSD test indicated that the ozone treatment group had significantly lower NIHSS scores and higher Barthel scores compared to the control and oxygen groups (*p* < 0.05) (Figure 1).

### 3.3 Comparison of cognitive function scores

**Figure 2:**
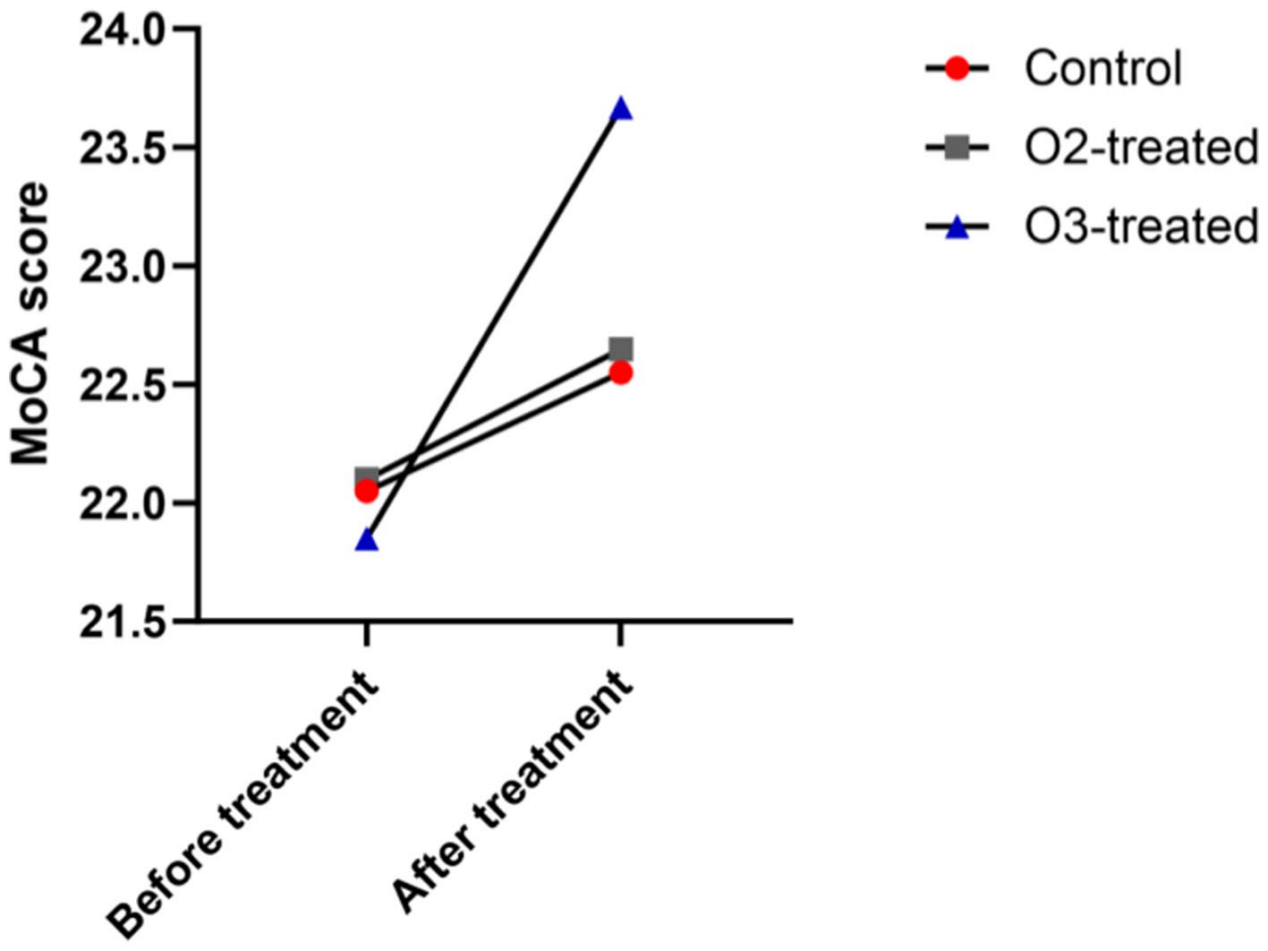
Comparison of cognitive function before and after treatment. Before treatment, there were no significant differences in the cognitive function (MoCA) scores (*p* > 0.05). After treatment, only the ozone therapy group showed a significant improvement in cognitive function compared to before treatment (*p* < 0.05).

Pre-treatment, no significant MoCA score differences existed among the three groups (*p* > 0.05). Post-treatment, the ozone group’s MoCA scores improved significantly (Cohen’s d = 0.7, *p* < 0.05), while the control and oxygen groups showed no significant changes (Cohen’s d = 0.2, *p* > 0.05). A one-way ANOVA was conducted to compare the MoCA scores across the three groups, revealing significant differences (*F* = 3.89, *p* < 0.05). *Post-hoc* analysis using Tukey’s HSD test indicated that the ozone treatment group had significantly higher MoCA scores compared to the control and oxygen groups (*p* < 0.05) (Figure 2).

### 3.4 Comparison of brain tissue injury indicators

**Figure 3:**
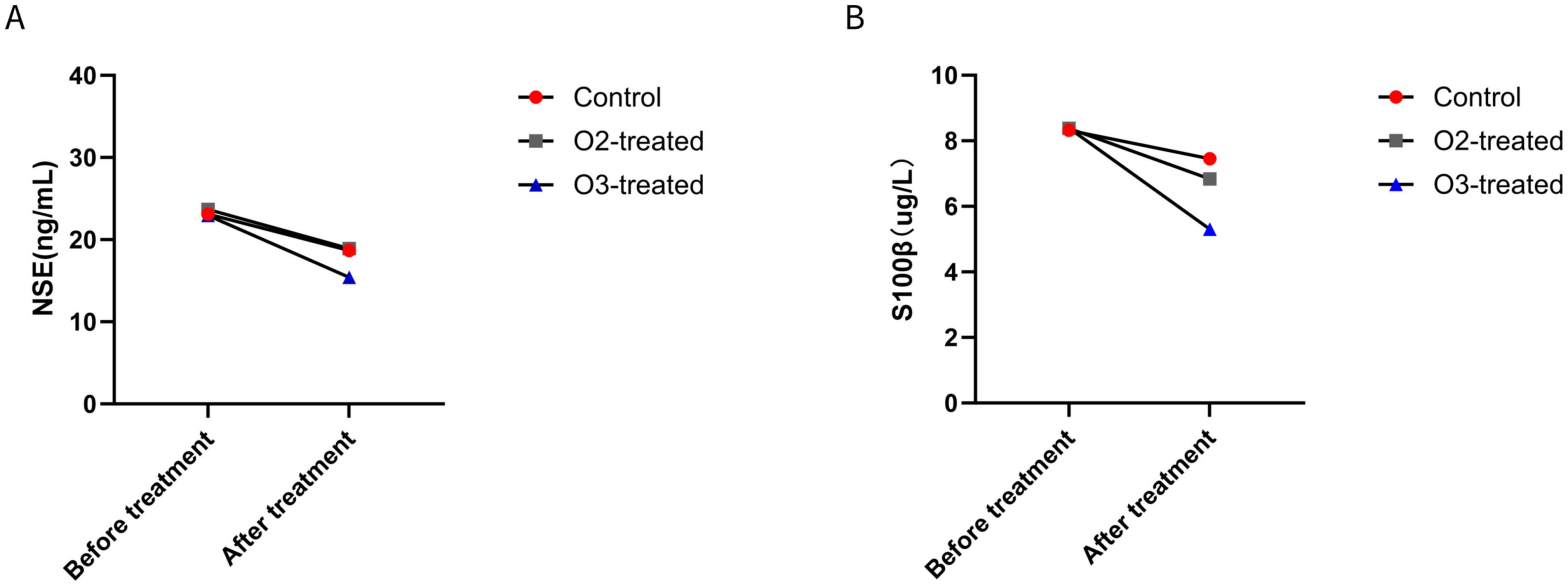
Comparison of brain tissue injury indicators before and after treatment. Before treatment, there were no significant differences in brain tissue injury indicators (*p* > 0.05). After treatment, only the ozone therapy group showed a significant improvement in NSE **(A)** and S100β **(B)** compared to before treatment (*p* < 0.05).

Pre-treatment, no significant differences in NSE and S100β levels existed among the three groups (*p* > 0.05). Post-treatment, the ozone group showed a significant decrease in NSE levels (Cohen’s d = 0.6, *p* < 0.05) and a significant reduction in S100β levels (Cohen’s d = 0.4, *p* < 0.05). Spearman correlation analysis revealed significant correlations between NIHSS scores and NSE/S100β levels (*ρ* = 0.65, *p* < 0.05), as well as between MoCA scores and S100β levels (*ρ* = −0.58, *p* < 0.05). For non-parametric data (NSE, S100β), the Wilcoxon signed-rank test was used for pre-post comparisons within groups, showing significant improvements in the ozone treatment group (*p* < 0.05) (Figure 3).

### 3.5 Oxidative stress-related indicators

**Figure 4:**
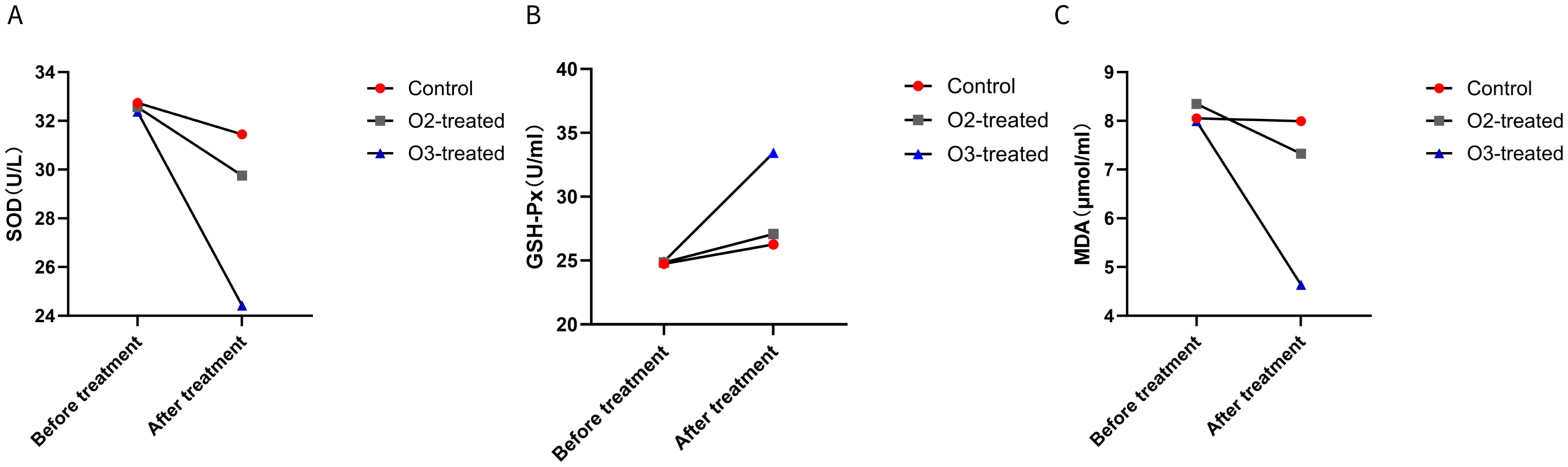
Comparison of oxidative stress markers before and after treatment. Before treatment, there were no significant differences in the levels of SOD **(A)**, GSH-Px **(B)**, and MDA **(C)** among the three groups (*P* > 0.05). After treatment, the ozone therapy group showed a significant decrease in SOD and MDA levels and a significant increase in GSH-Px levels compared to before treatment (*P* < 0.05). The control group showed no significant changes in the three markers compared to before treatment (*P* > 0.05). The oxygen therapy group showed no significant changes in SOD levels compared to before treatment, a significant increase in GSH-Px levels, and a significant decrease in MDA levels (*P* < 0.05). The magnitude of changes in the markers for the oxygen therapy group was lower than that of the ozone therapy group (*P* < 0.05).

Pre-treatment, no significant differences in SOD, GSH-Px, and MDA levels existed among the three groups (*p* > 0.05). Post-treatment, the ozone group demonstrated a significant decrease in SOD and MDA levels and a significant increase in GSH-Px levels (Cohen’s d = 0.5, *p* < 0.05). For non-parametric data (SOD, GSH-Px, MDA), the Wilcoxon signed-rank test was used for pre-post comparisons within groups, showing significant improvements in the ozone treatment group (*p* < 0.05) (Figure 4).

### 3.6 Comparison of hypoxia-inducible factors

**Figure 5:**
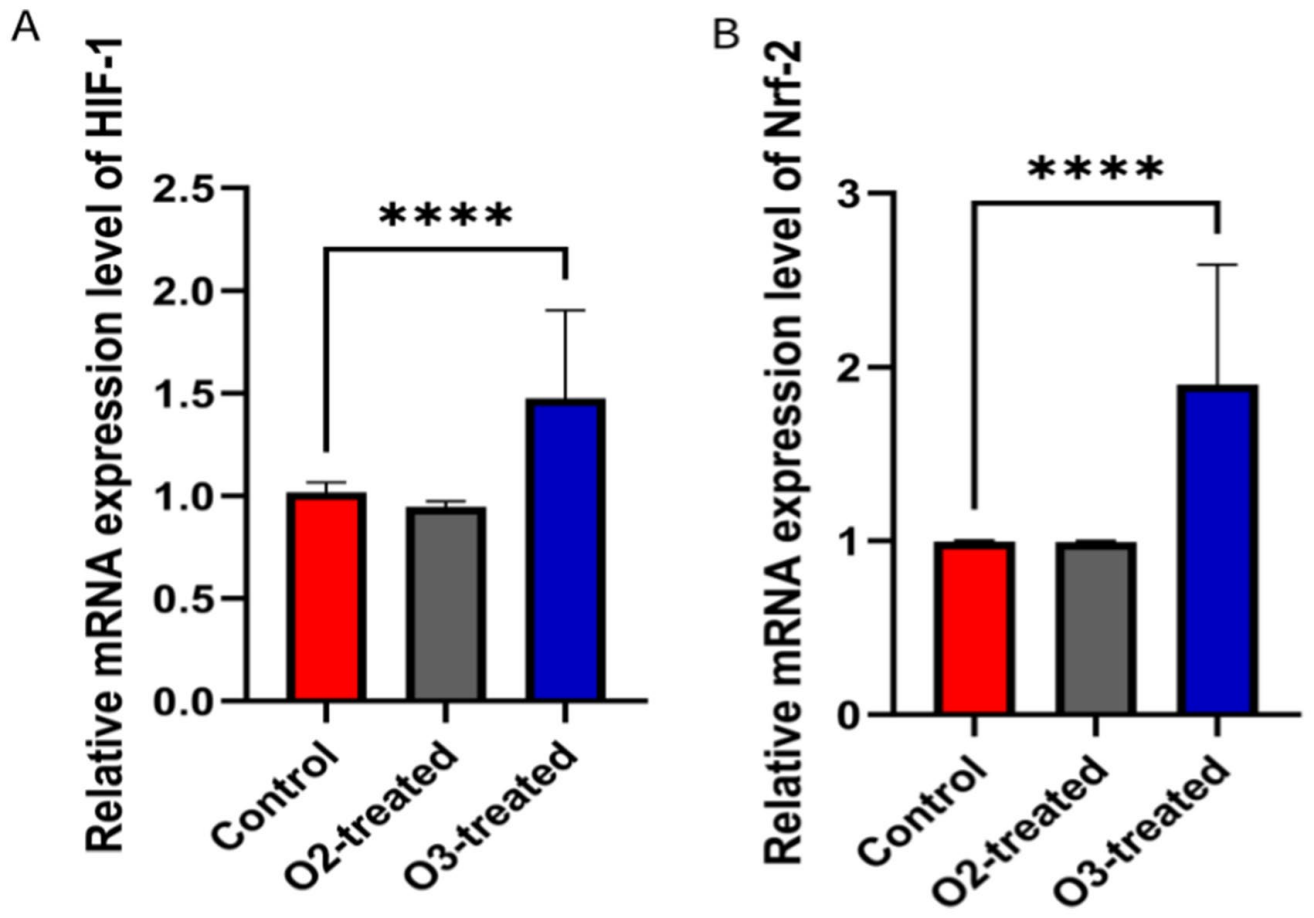
Comparison of hypoxia-inducible factors levels before and after treatment. Before treatment, there were no significant differences in HIF-1 and Nrf-2 levels among the three groups (*p* > 0.05). After treatment, the ozone therapy group showed a significant increase in HIF-1 **(A)** and Nrf-2 **(B)** levels compared to before treatment (*p* < 0.05), while the levels in the other two groups showed no significant changes compared to before treatment (*p* > 0.05).

Pre-treatment, no significant differences in HIF-1 and Nrf-2 levels existed among the three groups (*p* > 0.05). Post-treatment, the ozone group showed a significant increase in HIF-1 and Nrf-2 levels compared to baseline (Cohen’s d = 0.6, *p* < 0.05). A one-way ANOVA was conducted to compare the HIF-1 and Nrf-2 levels across the three groups, revealing significant differences (*F* = 5.23, *p* < 0.05). *Post-hoc* analysis using Tukey’s HSD test indicated that the ozone treatment group had significantly higher HIF-1 and Nrf-2 levels compared to the control and oxygen groups (*p* < 0.05) (Figure 5).

### 3.7 Safety-related indicators

**Figure 6:**
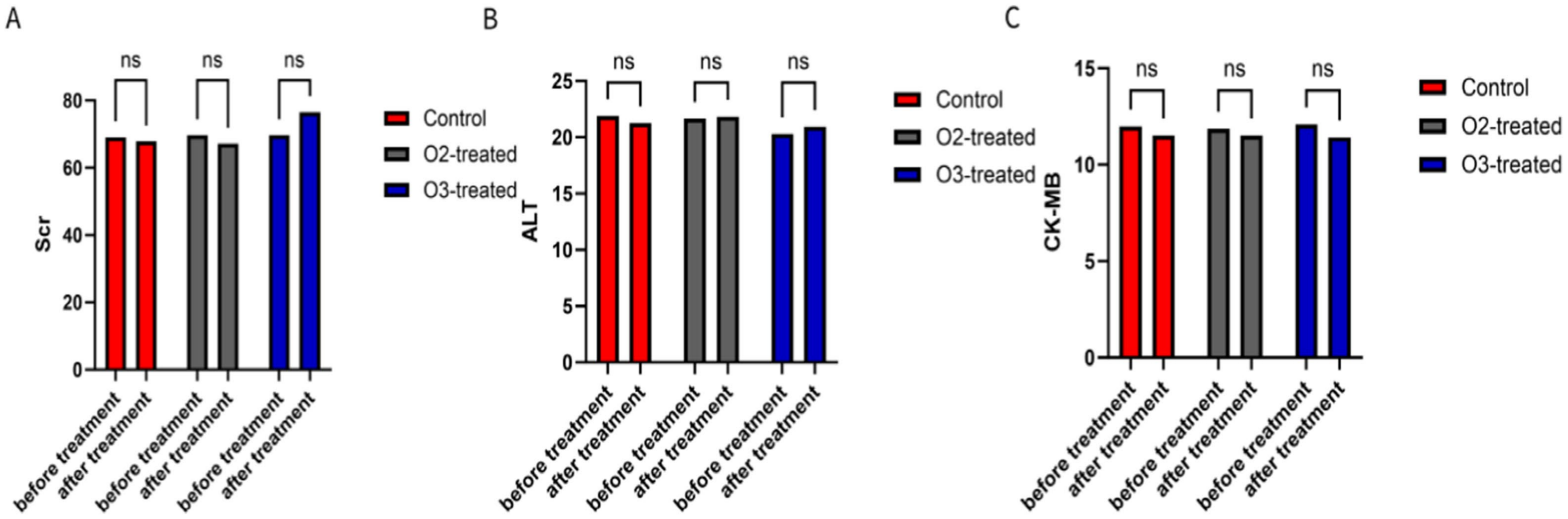
Comparison of safety-related indicators. Serum creatinine (Scr) **(A)**, Glutamic-pyruvic transaminase (ALT) **(B)**, and Creatine Kinase Isoenzyme (CK-MB) **(C)** were selected as indicators to assess renal function, liver function, and myocardial injury, respectively. There were no significant differences in these three indicators between the three groups of patients at the beginning and end of treatment (*p* > 0.05).

During the trial, six patients in the oxygen group and eight in the ozone group developed subcutaneous ecchymosis at the puncture site, which resolved naturally within a week without significant adverse events. Liver, kidney, and myocardial function indicators showed no significant pre- and post-treatment differences among the three groups (*p* > 0.05) (Figure 6). Long-term safety follow-up results (3 months) showed no significant differences in hemorrhagic transformation or infections among the three groups.

### 3.8 Comparison of clinical efficacy

**Figure 7:**
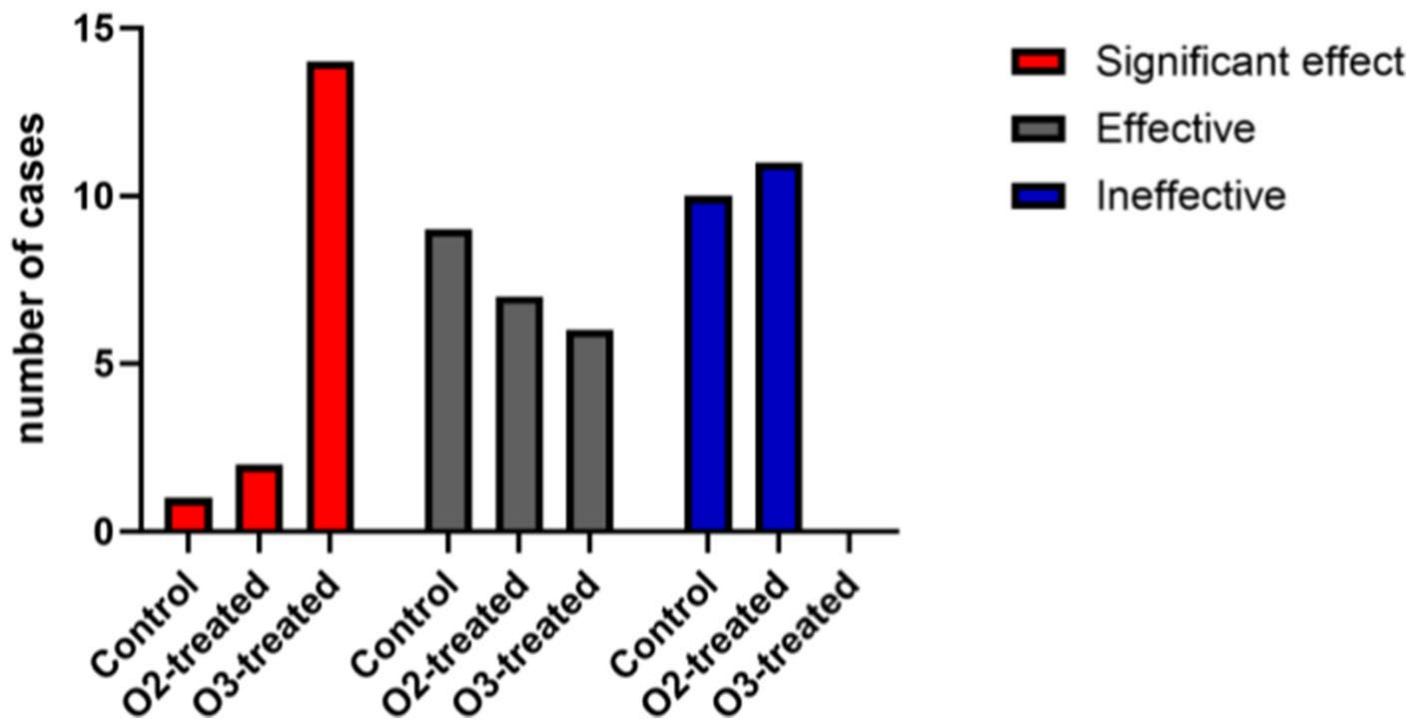
Comparison of clinical efficacy among the three treatment groups. The ozone treatment group showed better treatment outcomes compared to the control group and the oxygen treatment group, with statistically significant differences (*p* < 0.05). When comparing the oxygen treatment group with the control group, the oxygen treatment group showed slightly better efficacy, with statistically significant differences.

Post-treatment, the ozone group showed superior clinical efficacy to the control and oxygen groups (*p* < 0.05). A one-way ANOVA was conducted to compare the clinical efficacy scores across the three groups, revealing significant differences (*F* = 6.12, *p* < 0.05). *Post-hoc* analysis using Tukey’s HSD test indicated that the ozone treatment group had significantly higher clinical efficacy scores compared to the control and oxygen groups (*p* < 0.05) (Figure 7).

## 4 Discussion

This study aimed to investigate the therapeutic effects of ozone autohemotherapy on limb movement and cognitive impairment in patients with acute ischemic stroke, exploring its potential mechanisms and comparing therapeutic efficacy and oxidative stress responses with oxygenation therapy. The findings aim to provide clinical guidance for treatment.

During the development of acute cerebral infarction, ischemic hypoxic injures brain tissue, leading to the rupture of glial cells and the subsequent release of S100*β* protein, a cytoplasmic marker of glial cells (44). Moreover, serum S100*β* protein levels in patients with acute ischemic stroke have been positively correlated with the degree of neurological impairment (45). In this study, neurofunctional assessments using NIHSS, Barthel Index, and MoCA demonstrated that the ozone therapy group exhibited significantly reduced NSE and S100*β*levels post-treatment, paralleling improvements in NIHSS, Barthel Index, and MoCA scores. These improvements were significantly greater than those observed in the control and oxygen therapy groups. Moreover, the ozone therapy group showed enhanced clinical efficacy, with a higher proportion of patients showing favorable prognosis and fewer exhibiting poor prognosis. These findings underscore the efficacy of ozone autohemotherapy in mitigating brain tissue damage and improving outcomes in patients with acute ischemic stroke.

This study delved into the underlying mechanisms of ozone autohemotherapy in treating acute ischemic stroke. Existing literature indicates that ozone primarily functions through its anti-oxidative stress response (35, 46–49). A study by Mehraban et al. (50) demonstrated the therapeutic effect of ozone autohemotherapy, attributing them to its interaction with various biological components. Upon dissolution in biological fluids, ozone rapidly reacts with a series of biomolecules and generates ROS and lipid ozonation products (LOPs), termed “ozone messengers.” Initially, hydrogen peroxide (H_2_O_2_) is predominantly produced, while LOPs like 4-hydroxynonenal (4-HNE), which have a longer half-life, exert delayed effects throughout the body via the bloodstream. Notably, H_2_O_2_ and 4-HNE emerge as the most relevant secondary messengers in the biological activity of O_3_ (39).

The antioxidant mechanisms of ozone involve the activation of nuclear factor erythroid-2 related factor 2 (Nrf2) (51–53). LOPs regulate the transcriptional expression of antioxidant response elements (ARE) by activating Nrf2, thereby enhancing the expression of enzymes such as SOD, catalase (CAT), heme oxygenase-1 (HO-1), glutathione (GSH), and GSH-Px (54–56). O_3_ also regulates casein kinase 2 (CK2) to phosphorylate and modulate Nrf2 activity, mitigating oxidative stress. In this study, the levels of SOD, GSH-Px, and MDA in patients treated with ozone autohemotherapy displayed significant alterations (57, 58). Additionally, Nrf2 levels were upregulated, further demonstrating that the process of ischemic vascular disease treatment with ozone involves regulating oxidative stress in acute cerebrovascular diseases by modulating the level of Nrf2.

Hypoxia-inducible factor-1 (HIF-1), a heterodimeric protein and a member of the basic helix–loop–helix family, plays a critical role in the body’s adaptive response to ischemic and hypoxic conditions (59, 60). Hypoxia-inducible factor-1 (HIF-1), a heterodimeric protein and a member of the basic helix–loop–helix family, plays a critical role in the body’s adaptive response to ischemic and hypoxic conditions (61). It also induces erythropoietin production, promoting red blood cell generation and increasing oxygen supply to tissues and cells, thus enhancing their tolerance to hypoxic environments (62). Furthermore, elevated HIF-1 expression is observed in cerebral infarction (63–65). Where it promotes glucose transport and consequently protects the ischemic and hypoxic neurons (66–69). This study found comparable baseline serum HIF-1 levels among the ozone therapy, control, and oxygen therapy groups before treatment (*p* > 0.05). However, post-treatment, HIF-1 levels significantly increased in the ozone therapy group while remaining unchanged in the control and oxygen therapy groups. These differences were statistically significant (*p* < 0.05), suggesting that ozone autohemotherapy enhances serum HIF-1 levels, potentially exerting a neuroprotective effect.

In this study, patients in both the ozone therapy and oxygen therapy groups experienced subcutaneous hematoma at the puncture site, which did not necessitate treatment withdrawal or lead to adverse effects. After 5 days of treatment, there were no statistically significant differences (*p* > 0.05) observed in liver and kidney function, as well as myocardial enzyme indicators, among the three groups. Furthermore, no drug-related adverse reactions or other adverse events, such as abnormalities in the liver, kidney, or cardiac enzyme, occurred during the study period. These findings suggest that ozone autohemotherapy is relatively safe and does not induce liver, kidney, or myocardial toxicity. It presents a viable treatment option for patients with acute ischemic stroke complicated by liver, kidney, and cardiac dysfunction (70, 71).

Ozone autohemotherapy demonstrates unique advantages compared to conventional antioxidant agents such as edaravone. Unlike edaravone, which exerts a singular antioxidant effect through free radical scavenging, ozone therapy simultaneously activates dual pathways involving Nrf2 and HIF-1. This dual activation not only effectively mitigates oxidative stress damage but also improves cerebral hypoxia. In terms of safety, ozone treatment did not exhibit the renal impairment risk associated with edaravone (reported incidence ~5%). These findings indicate that ozone therapy, through its multi-target synergistic mechanism, outperforms traditional antioxidant drugs in both neurological recovery and safety profile, offering a novel therapeutic alternative for acute ischemic stroke.

This study is a clinical trial with many confounding factors that could influence the results. All patients received standard treatments including aspirin for antiplatelet aggregation and rosuvastatin for lipid-stabilizing plaque medication to minimize potential variation in treatment outcomes. However, further research is warranted. To mitigate the impact of oxygen dissolution from ozone decomposition into the blood, an oxygen therapy group was included as a control, demonstrating that the therapeutic effects of ozone autohemotherapy are distinct from those of oxygen therapy. In summary, ozone autohemotherapy demonstrates clear clinical efficacy in treating acute ischemic stroke by enhancing cognitive and motor functions, facilitating neurological recovery, and maintaining a high safety profile. It suggests that ozone may exert its therapeutic effects through modulation of the Nrf2 and HIF-1 pathways, potentially mitigating oxidative stress injury and demonstrating possible protective effects against ischemia-hypoxia conditions. This study provides a certain degree of basis and guidance for the future clinical application of ozonated autohemotherapy. However, the sample size in this study is relatively small, and the research on the pathways is not sufficiently in-depth. In the future, we will focus on the controversies surrounding the use of ozonated autohemotherapy in the treatment of acute ischemic stroke. Increasing the sample size will make the research results more convincing, and we will further investigate the mechanisms of the pathways based on the results of this study. Evaluating various concentrations of medical ozone will provide comprehensive insights into treatment efficacy, benefits, potential adverse reactions, and organ function preservation. Considering external factors and optimizing combination therapy will ensure the reliability and safety of ozone autohemotherapy in clinical practice.

## Data Availability

The raw data supporting the conclusions of this article will be made available by the authors, without undue reservation.
